# Evaluation of Iron Oxide Nanoparticle Micelles for Magnetic Particle Imaging (MPI) of Thrombosis

**DOI:** 10.1371/journal.pone.0119257

**Published:** 2015-03-06

**Authors:** Lucas W. E. Starmans, Rik P. M. Moonen, Erica Aussems-Custers, Mat J. A. P. Daemen, Gustav J. Strijkers, Klaas Nicolay, Holger Grüll

**Affiliations:** 1 Biomedical NMR, Department of Biomedical Engineering, Eindhoven University of Technology, Eindhoven, the Netherlands; 2 Applied Chemistry, Philips Research, Eindhoven, the Netherlands; 3 Department of Pathology, Academic Medical Center, Amsterdam, the Netherlands; 4 Biomedical Engineering and Physics, Academic Medical Center, Amsterdam, the Netherlands; 5 Oncology Solutions, Philips Research, Eindhoven, the Netherlands; Baker IDI Heart and Diabetes Institute, AUSTRALIA

## Abstract

Magnetic particle imaging (MPI) is an emerging medical imaging modality that directly visualizes magnetic particles in a hot-spot like fashion. We recently developed an iron oxide nanoparticle-micelle (ION-Micelle) platform that allows highly sensitive MPI. The goal of this study was to assess the potential of the ION-Micelles for MPI-based detection of thrombi. To this aim, an *in vivo* carotid artery thrombosis mouse model was employed and *ex vivo* magnetic particle spectrometer (MPS) measurements of the carotid arteries were performed. In addition, we studied the effect of functionalization of the ION-Micelle nanoplatform with fibrin-binding peptides (FibPeps) with respect to nanoparticle thrombus uptake and hence thrombus detection. *In vivo* quantitative MR imaging pre- and post-ION-Micelle injection was performed as reference for visualization of ION-micelle uptake. ION-Micelles significantly decreased T_2_ values in the thrombi with respect to pre-injection T_2_ values (p < 0.01) and significantly increased *ex vivo* MPS thrombus signal with respect to the noninjured, contralateral carotid (p < 0.01). Functionalization of the ION-Micelles with the FibPep peptides did not result in an increased MPS thrombus signal with respect to the non-fibrin binding ION-Micelles. The lack of a significant increased thrombus uptake for the FibPep-ION-Micelles indicates that (non-fibrin-specific) entrapment of nanoparticles in the mesh-like thrombi is the key contributor to thrombus nanoparticle uptake. Therefore, (nontargeted) ION-Micelles might be of value for noninvasive MPI-based diagnosis, characterization and treatment monitoring of thrombosis.

## Introduction

Thrombi play an important role in multiple cardiovascular diseases, such as deep vein thrombosis, pulmonary embolism, ischemic stroke and myocardial infarction. Currently, detection of thrombi entails a multitude of imaging modalities, including MRI, coronary angiography, transesophageal echocardiography, carotid and pelvic ultrasound, ventilation-perfusion scanning and computed tomography (CT), each characterized by its own set of strengths and weaknesses [1–6]. A noninvasive “one stop shop” methodology for thrombosis imaging would therefore be a welcome addition to the current armature of thrombus diagnostic strategies.

Magnetic particle imaging (MPI) is a relatively new diagnostic imaging modality that is able to directly visualize magnetic nanoparticles [7]. This represents a fundamentally different approach than iron oxide nanoparticle-enhanced MRI, which detects magnetic nanoparticles indirectly by measuring their effect on proton relaxation rates. MPI yields hotspot-like images similar to nuclear imaging techniques and has the potential to provide a “one stop shop” methodology for thrombosis diagnosis. Nanoparticles accumulate in thrombi by entrapment in the thrombus mesh and this phenomenon has previously been employed for thrombosis diagnostic purposes using iron oxide nanoparticles for MRI-aided diagnosis [8] and gold nanoparticles for CT-aided diagnosis [9].

Recently, we have developed an iron oxide nanoparticle micelle (ION-Micelle) platform that allows sensitive MPI [10]. The ION-Micelle nanoplatform consists of hydrophobic, 25 nm-sized iron oxide nanoparticles, which are encapsulated into pegylated phospholipid micelles. For optimal sensitivity in MPI, the nanocrystal size of the iron oxide nanoparticles should be around 20–30 nm [11], whereas currently employed commercially available iron oxide formulations for MPI have a core size around 4–6 nm [12,13]. This size is too small to induce a strong signal for MPI. Magnetic particle spectrometry (MPS) [14], which essentially is zero-dimensional MPI, showed that ION-Micelles induced up to 200-fold higher signal compared to Resovist [10], which is considered to be the most potent commercially available iron oxide formulation for MPI purposes. Thus, the ION-Micelle nanoplatform allows significantly more sensitive MPI than current commercially available iron oxide formulations. Due to their strong T_2_ effect [10], these particles can also be employed for sensitive MRI detection, which allows a direct comparison of MPI and MRI-based detection.

In this study, we assessed the potential of the ION-Micelle nanoplatform for nanoparticle entrapment-based detection of thrombi using MPI. To this aim, mice with induced carotid artery thrombosis were injected with the ION-Micelles and *ex vivo* MPS measurements of the carotid arteries were obtained. *In vivo* MRI of the thrombi pre- and post-ION-Micelle injection was performed as a reference methodology. Available options for visualizing thrombi with MRI include non-contrast-enhanced methods such as T_1_-weighted imaging [15]. However, because of the use of ION-micelles in this study, a T_2_-mapping approach was chosen to visualize the uptake of these particles in a sensitive fashion.

Additionally, this study investigates whether functionalization of the ION-Micelle nanoplatform with fibrin-binding peptides (FibPep) yields an increase in thrombus uptake of the nanoparticles, and thus improves thrombosis diagnosis by combining nanoparticle entrapment and fibrin-binding as nanoparticle thrombus uptake mechanisms. FibPep contains the cyclic fibrin-binding amino acid sequence RWQPCPAESWT-Cha-CWDP and binds to human and mouse fibrin with an affinity (K_d_) of approximately 700 nM [16]. As a negative control with respect to fibrin-binding, ION-Micelles were functionalized with a scrambled version of FibPep (NCFibPep). Previous preliminary *in vitro* fibrin-binding experiments showed that FibPep-ION-Micelles bound significantly more to human plasma clots than NCFibPep-ION-Micelles and this increased uptake could be visualized and quantified using MRI and MPS, respectively [10]. In this study, we performed more elaborate *in vitro* characterization and additionally assessed whether such a fibrin-binding strategy also leads to increased thrombus nanoparticle uptake *in vivo*.

## Results

### Nanoparticle synthesis and characterization

**Fig 1:**
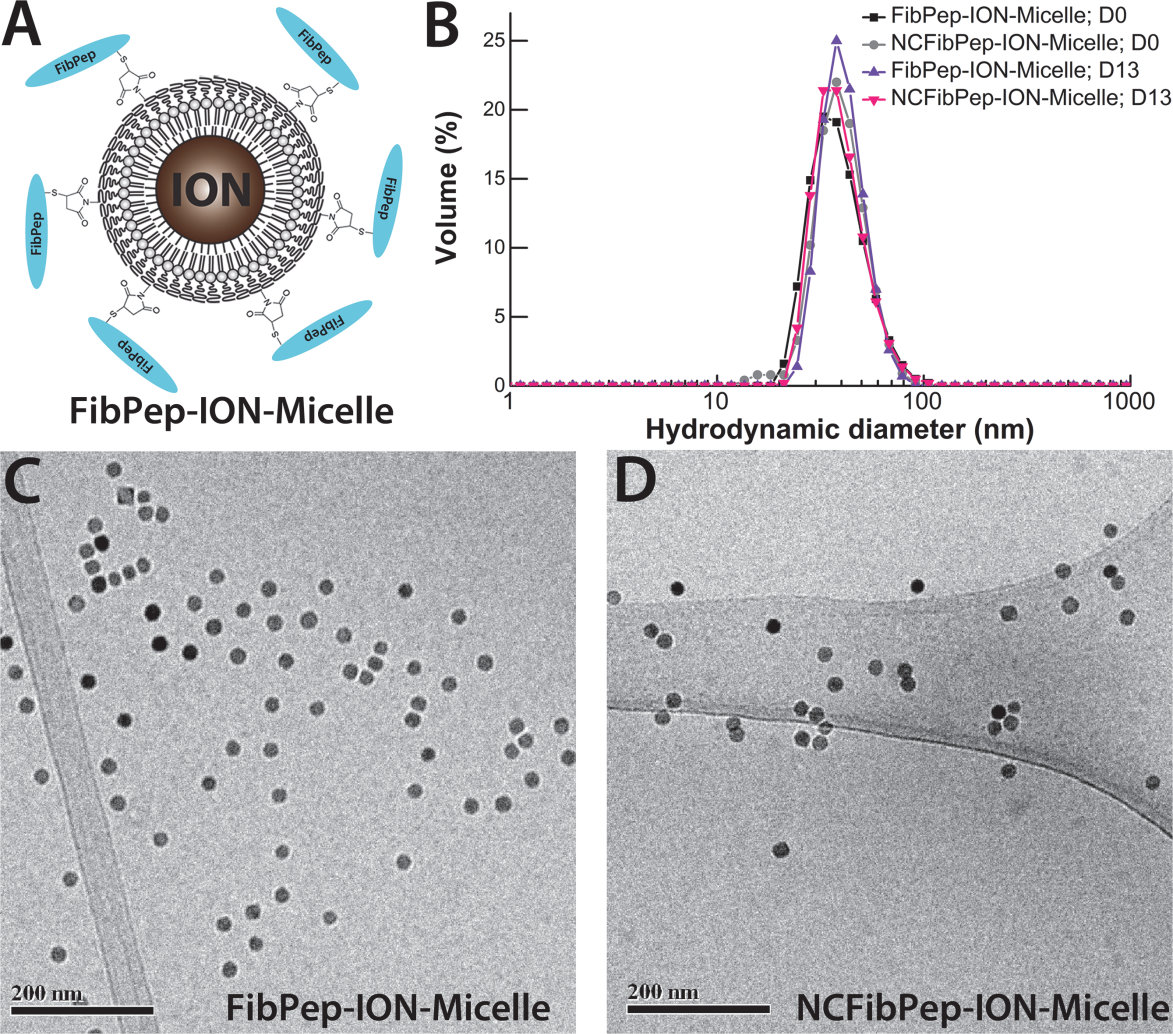
FibPep-ION-Micelle nanoplatform. (A) Schematic representation of the FibPep-ION-Micelle nanoplatform. Reproduced from Starmans and coworkers [10]. (B) Volume-weighted size-distribution profiles of the FibPep-ION-Micelles and NCFibPep-ION-Micelles at the day of synthesis (D0) and at the final day of the *in vivo* experiments (13 days post synthesis, D13). (C, D) Representative cryo-TEM images of (C) FibPep-ION-Micelles and (D) NCFibPep-ION-Micelles.

**Fig 2:**
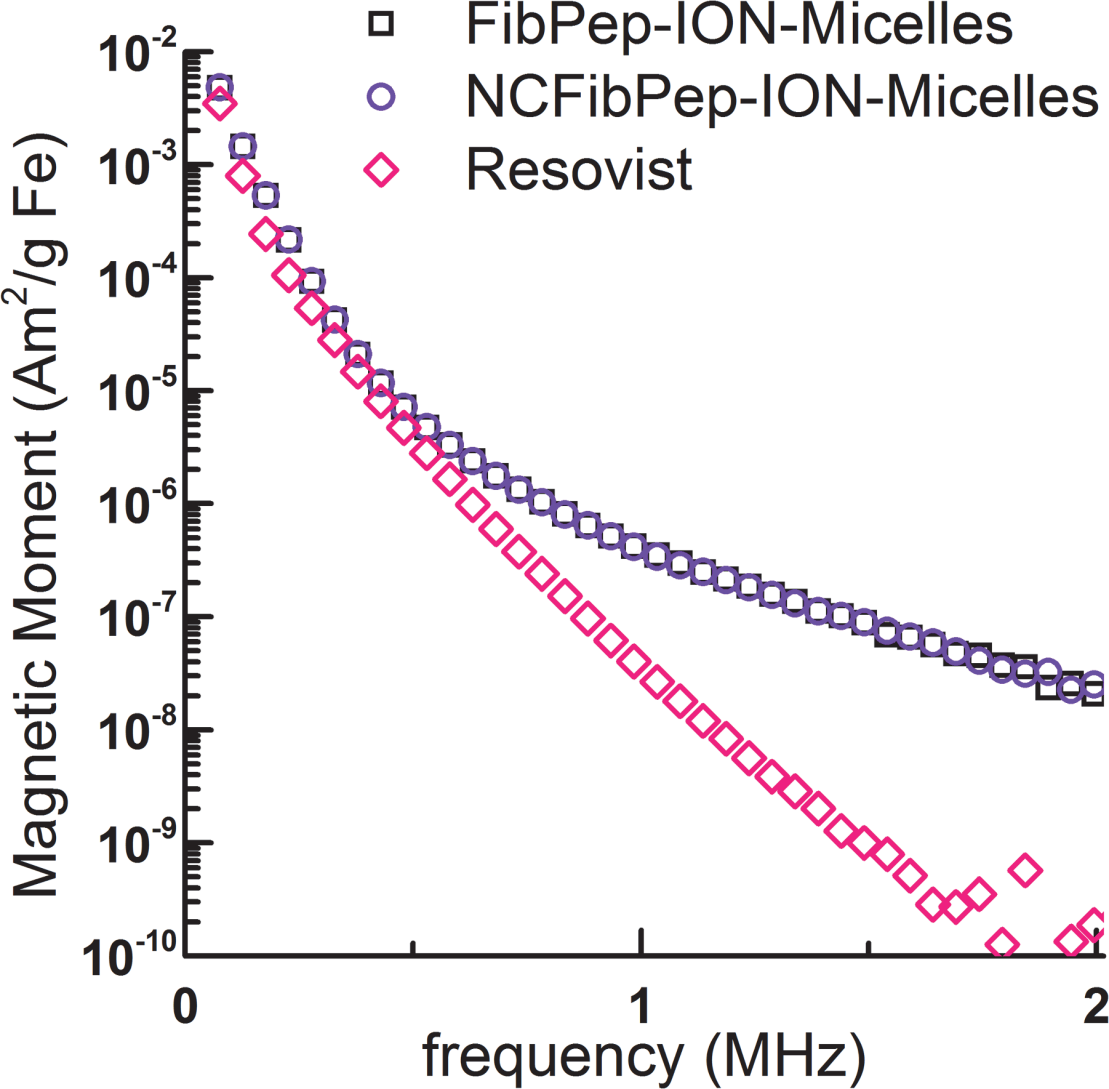
MPS of FibPep-ION-Micelles, NCFibPep-ION-Micelles and Resovist. Data is plotted as magnetic moment (normalized for iron content) versus frequency. Note that the curves of FibPep-ION-Micelles and NCFibPep-ION-Micelles largely overlap.

Fibrin-targeted FibPep-ION-Micelles (Fig. 1A) and non-fibrin-specific NCFibPep-ION-Micelles were synthesized according to a previously published protocol [10]. The dispersion state of the synthesized nanoparticles in HEPES buffered saline (HBS, pH 7.4) was investigated using dynamic light scattering (DLS) and cryogenic transmission electron microscopy (cryo-TEM) measurements. Immediately after synthesis of the nanoparticles, one peak was observed with a maximum intensity at a hydrodynamic diameter of 40 nm (Fig. 1B). At the conclusion of the *in vivo* experiments (13 days post-synthesis), DLS was performed once more, and showed identical results for both FibPep-ION-Micelles and NCFibPep-ION-Micelles, indicating excellent intrinsic stability of the particles over the time course of the study. Cryo-TEM analysis showed that FibPep-ION-Micelles and NCFibPep-ION-Micelles were dispersed in HBS as single particles or as small aggregates of nanoparticles (Fig. 1C-D). FibPep-ION-Micelles and NCFibPep-ION-Micelles displayed a longitudinal relaxivity of 5.6 and 5.4 mM^-1^s^-1^ and a transversal relaxivity of 207 and 204 mM^-1^s^-1^, respectively and are thus well suited to allow sensitive detection by T_2_-weighted MR imaging. MPS was performed to assess the capacity of the (NC)FibPep-ION-Micelles to induce a signal for MPI purposes. Resovist was measured as a reference. FibPep-ION-Micelles induced similar MPS signal in comparison to NCFibPep-ION-Micelles (Fig. 2). The signal of (NC)FibPep-ION-Micelles was up to 750 times increased with respect to Resovist, indicating that the (NC)FibPep-ION-Micelles are potent contrast agents for MPI.

An *in vitro* human blood clot assay was performed to confirm the specific fibrin-binding capabilities of the synthesized batch of FibPep-ION-Micelles. Plasma clots were incubated with (NC)FibPep-ION-Micelles and extensively washed after incubation. Subsequently, clots were photographed and measured using MPS to determine nanoparticle binding. The clots incubated with FibPep-ION-Micelles showed markedly increased uptake of the brownish colored ION-Micelles with respect to NCFibPep-ION-Micelles incubated clots (S1A Fig). Specific analysis of the third MPS harmonic (72.6 kHz) showed that FibPep-ION-Micelles displayed a 3-fold higher signal in the clots compared to NCFibPep-ION-Micelles (S1B Fig). Thus, FibPep-ION-Micelles bound in specific fashion to the clots. Samples were stored at 4°C and measured once more after more than 1.5 years of storage, yielding virtually identical results (S1B Fig), indicating that the particles were bound to the clots in a stable fashion.

### In vivo MRI

To study the potential of the ION-Micelles to allow visualization of thrombi using MPI, an AlCl_3_-induced carotid artery thrombosis mouse model was employed [17]. *In vivo* MRI of the neck region was performed as a reference methodology. The AlCl_3_-model produces wall-adherent thrombi similar to the frequently employed FeCl_3_-injury method [16,18,19], but does not cause iron-based MR signal void artifacts which hamper MRI analysis of the produced thrombi using the FeCl_3_-model [17]. Mice were subjected to baseline MR scans following thrombus inducing surgery, and, subsequently, FibPep-ION-Micelles or NCFibPep-ION-Micelles were injected and mice underwent post-injection MR scans (n = 5 per group). No adverse effects were noticed following injection of the nanoparticles. One mouse of the NCFibPep-ION-Micelle group had to be excluded from MRI data analysis because of internal bleeding caused by the surgery, obscuring the carotid artery and thrombus in the MR images.

**Fig 3:**
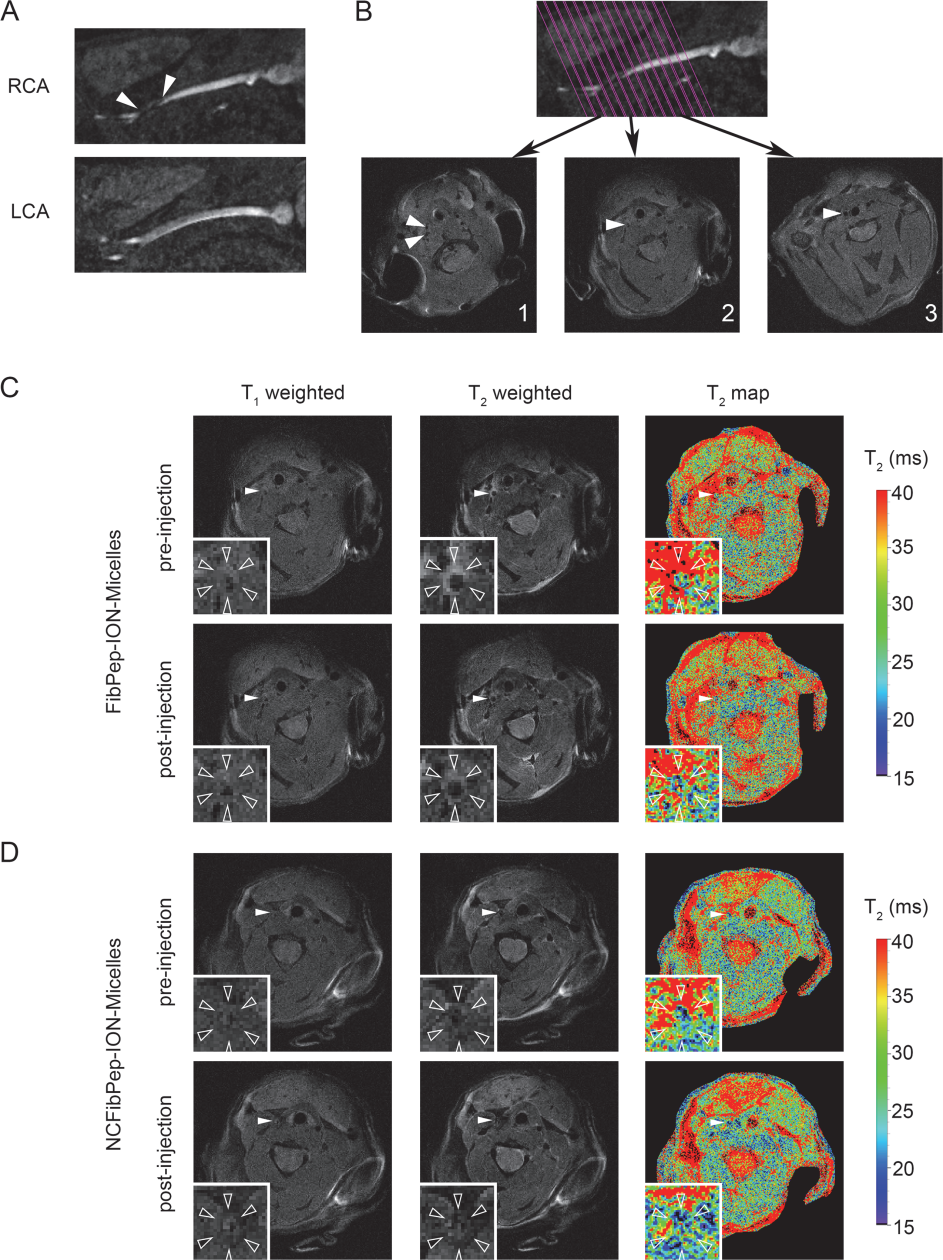
*In vivo* MRI. (A) Images of right and left carotid arteries (RCA & LCA) reconstructed from the 3D time of flight (TOF) image. The thrombus can be observed in the RCA just proximal of the bifurcation (arrowheads). Presence of blood flow in the thrombosed carotid was confirmed for all animals. (B) On the TOF image 13 parallel slices were planned perpendicular to the RCA. T_1_-weighted images from three slices are shown: (1) distal from the bifurcation, (2) in the thrombus and (3) proximal to the thrombus (arrowheads: RCA). (C, D) Pre- and post-injection T_1_- and T_2_-weighted images and T_2_ maps of (C) FibPep-ION-Micelles and (D) NCFibPep-ION-Micelles (arrowheads: RCA). The insets show magnifications of the RCA (open arrowheads: outer vessel wall).

**Fig 4:**
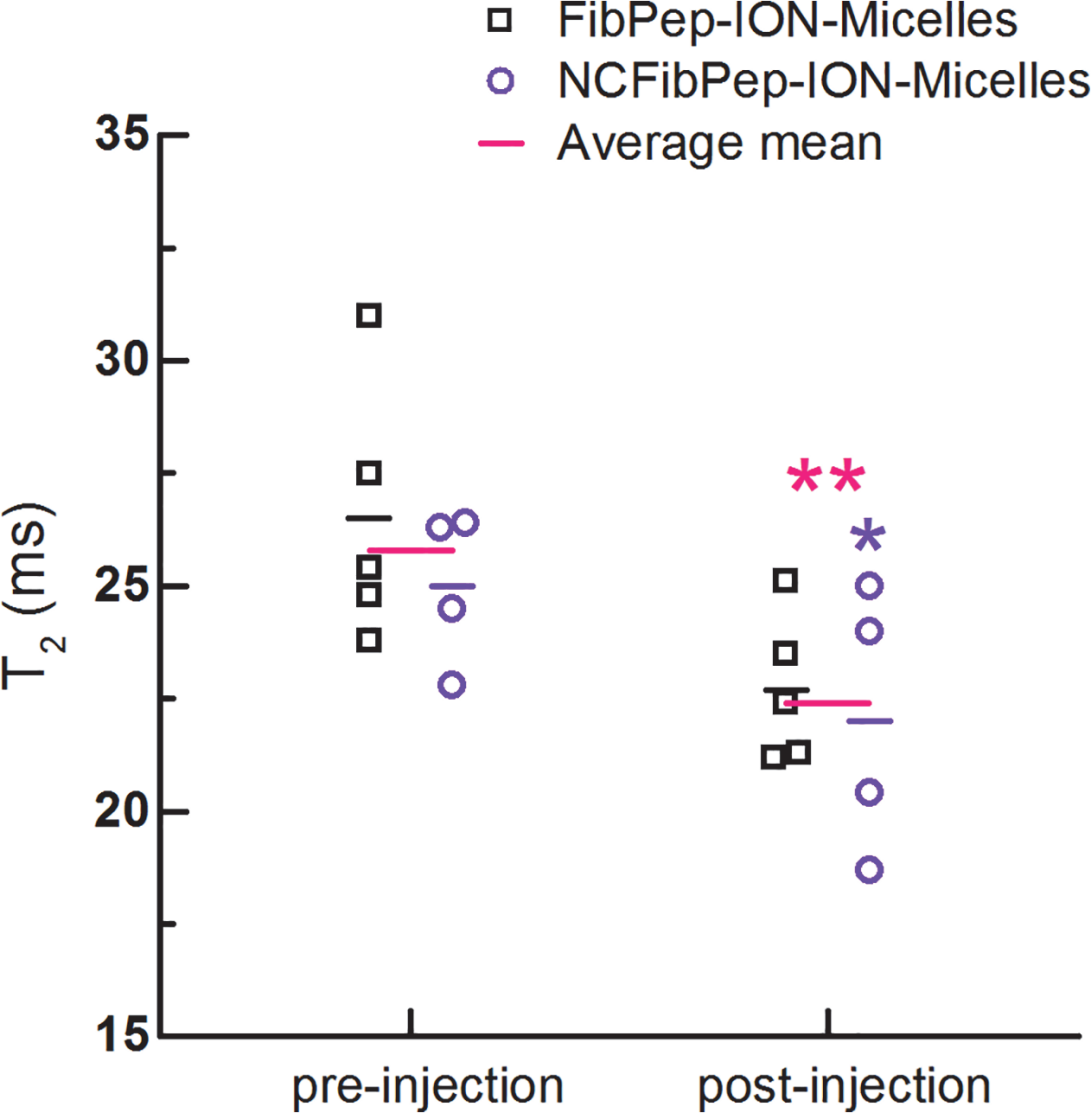
Quantitative analysis *in vivo* MRI scans. *In vivo* T_2_ of the thrombus pre- and post-injection with FibPep-ION-Micelles (n = 5) and NCFibPep-ION-Micelles (n = 4). The horizontal lines represent the group means and the average mean for the two groups combined (n = 9). Mean T_2_ values were significantly decreased post-injection compared to pre-injection for the NCFibPep-ION-Micelles group (p < 0.05; marked with *) and combined group (p < 0.01; marked with **).

3D fast low-angle shot time-of-flight (3D-FLASH-TOF) MRI images confirmed formation of thrombus in the right carotid artery, which was observed as interruption of the bright blood signal; the thrombus itself has a light gray appearance in the MR image (Fig. 3A). A 2D image of the right carotid artery (RCA) was reconstructed from the 3D dataset and was used for planning subsequent MRI scans (Fig. 3B). T_1_- and T_2_-weighted images and T_2_ maps were successfully acquired repeatedly (Fig. 3C-D). Quantification of T_2_ was preferred over T_2_* because imaging in the carotid artery region requires a protocol that is robust for cardiac, respiratory and blood-flow motion. Region of interest (ROI) analysis revealed decreased mean T_2_ values in the thrombus area after injection with both FibPep-ION-Micelles (22.7 ± 1.5 ms) and NCFibPep-ION-Micelles (22.0 ± 2.6 ms) compared to pre-injection values (26.5 ± 2.6 and 25.0 ± 1.5 ms, respectively) (Fig. 4). Comparison of T_2_ values between mice injected with FibPep-ION-Micelles or NCFibPep-ION-Micelles revealed no significant differences for pre- or post-injection values (p = 0.388 and p = 0.675, respectively). These findings indicate that the ION-Micelles accumulate in the thrombi and that the functionalization with FibPep did not specifically enhance *in vivo* uptake of ION-Micelles in the thrombus as compared to the non-fibrin-binding nanoparticles. Statistically, the mice injected with FibPep-ION-Micelles or NCFibPep-ION-Micelles can thus be viewed as a single group. Comparison of the average mean thrombus T_2_ values revealed a highly significant (p = 0.003) overall decrease of T_2_ from 25.8 ± 2.3 ms pre-injection to 22.4 ± 2.1 ms post-injection of ION-Micelles (Fig. 4).

### *Ex vivo* MPS and histological validation

**Fig 5:**
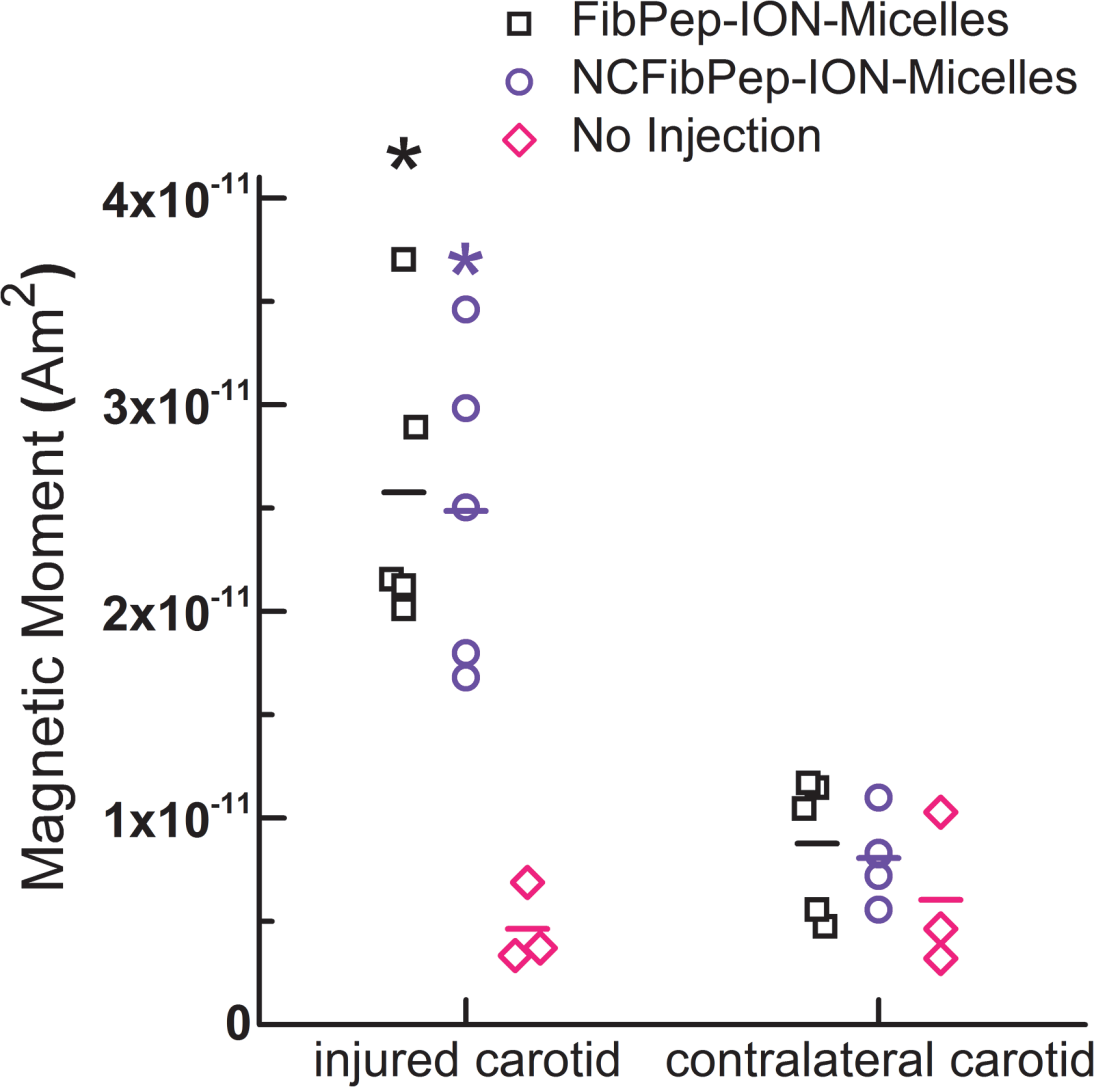
*Ex vivo* MPS of carotid arteries. MPS measurements of excised injured and contralateral, noninjured carotid arteries following injection with FibPep-ION-Micelles (n = 5) and NCFibPep-ION-Micelles (n = 5) or without injection (n = 3). Data is expressed as magnetic moment of the third harmonic (76 kHz), horizontal lines represent group means. * P < 0.01 versus contralateral carotid (all three groups) and injured carotid of mice which had not received an injection of ION-Micelles.

Mice were euthanized upon completion of the MR scans (ca. 2 h post-injection of ION-Micelles and 4.5 h post-thrombus induction). Subsequently, the injured and noninjured, contralateral carotid arteries were excised and measured using MPS to probe ION-Micelle MPI signal. In addition, the injured and contralateral carotids of three mice that had not undergone nanoparticle injection were measured with MPS to quantify background MPS signal of the thrombosed and noninjured carotids. Specific analysis of the third MPS harmonic (Fig. 5) showed significantly increased signal for the injured carotids of mice injected with FibPep-ION-Micelles or NCFibPep-ION-Micelles (26 ± 7 and 25 ± 8 pAm^2^, respectively) with respect to the contralateral carotids of these mice (9 ± 3 and 8 ± 2 pAm^2^, respectively) and also with respect to both the injured and contralateral carotids of mice which did not receive nanoparticle injections (5 ± 2 and 6 ± 4 pAm^2^, respectively). Thus, the ION-Micelles showed significant accumulation in thrombi. The MPS data indicate that there was no significant difference between iron oxide nanoparticle uptake in the injured carotids of mice injected with FibPep-ION-Micelles or NCFibPep-ION-Micelles, which is in line with the above MRI findings.

**Fig 6:**
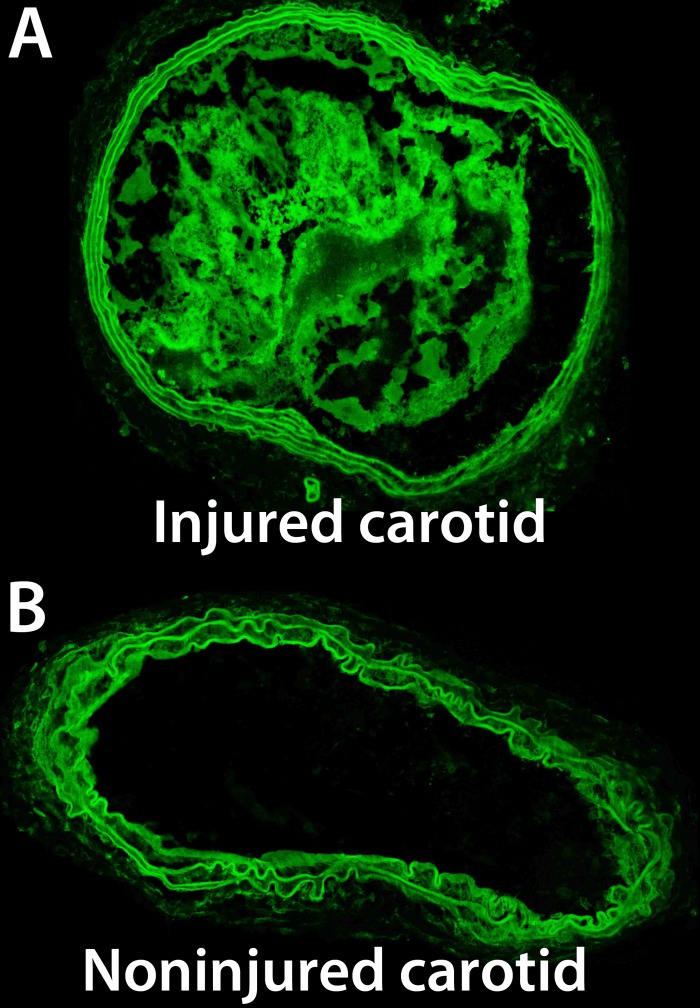
*Ex vivo c*onfocal microscopy of carotid arteries. Representative autofluorescence confocal microscopy images of transversal histological sections of the (A) injured carotid and (B) noninjured, contralateral carotid artery.

Histological sections studied using autofluorescence imaging confirmed (partial) occlusion of the injured carotid arteries and absence of occlusion in the contralateral, noninjured carotid arteries (Fig. 6).

## Discussion

In this study, we report on the characterization and the *in vivo* evaluation of the ION-Micelle nanoplatform for MPI-based detection of thrombi. FibPep-ION-Micelles and non-fibrin-binding NCFibPep-ION-Micelles had a volume-weighted hydrodynamic diameter of 40 nm and displayed excellent stability over time. The nanoparticles showed high transversal relaxivity (> 200 mM^−1^s^−1^) and high signal in magnetic particle spectroscopy (MPS) experiments (up to 750 fold increased signal compared to Resovist), indicating that the (NC)FibPep-ION-Micelles are potent contrast agents for MRI and MPI purposes. These characteristics of the FibPep-ION-Micelles and NCFibPep-ION-Micelles are virtually identical compared to (non-peptide functionalized) ION-Micelles [10]. The FibPep-ION-Micelles displayed significantly more binding towards blood clots *in vitro* with respect to negative control peptide (NCFibPep) functionalized ION-Micelles. These results were in line with previously reported findings [10]. In addition, we also assessed the binding stability over a longer period of time. The FibPep-ION-Micelles did not dissociate in a significant fashion from the blood clots after more than 1.5 year of incubation in buffer solution, indicating highly stable bonding between the nanoparticles and fibrin deposited in the blood clots *in vitro*.

To evaluate the potential of the ION-Micelle nanoplatform for MPI-based thrombus detection, an AlCl_3_-induced carotid artery thrombosis mouse model was employed. MR imaging of the carotids was performed pre- and post-nanoparticle injection as a reference methodology for visualization of ION-Micelle uptake. Pre-injection 3D-TOF images confirmed carotid artery thrombosis, whereas no signal void artifacts were observed, indicating that the AlCl_3_-injury method induced carotid artery thrombosis without inducing metal-based signal loss. Both FibPep-ION-Micelles and NCFibPep-ION-Micelles decreased the T_2_ values in the thrombus region, indicating that the ION-Micelles accumulated in significant fashion in the thrombi. No significant differences for pre- or post-injection values between the FibPep-ION-Micelle group and the group which received NCFibPep-ION-Micelles were observed, suggesting that the fibrin-binding peptides did not significantly contribute to the nanoparticle thrombus uptake *in vivo*. Hence, the observed thrombus uptake of the ION-Micelles is likely mainly due to nanoparticle entrapment in the mesh-like thrombi [8,9]. Previous *in vitro* MRI evaluation demonstrated that particle binding occurs only on the outside of the blood clots [10]. For reasons of resolution, *in vivo* ROIs encompass the whole thrombus, the vessel wall and potentially some blood, and local changes are thus averaged out over a larger area at the cost of sensitivity. After the final MR scans, mice were euthanized and the carotids were excised and measured *ex vivo* with a magnetic particle spectrometer. The FibPep-ION-Micelle-group and NCFibPep-ION-Micelle-group both displayed increased MPS signal in the injured carotid with respect to the noninjured, contralateral carotid artery. Thus, the ION-Micelles yield significant thrombus-specific signal for MPI purposes. No significant difference between FibPep-ION-Micelles and non-fibrin-binding NCFibPep-ION-Micelles was observed, which corresponds well with the *in vivo* MR imaging results.

The lack of significant additional thrombus uptake for the fibrin-binding FibPep-ION-Micelles with respect to non-fibrin binding NCFibPep-ION-Micelles *in vivo* may have various potential causes. First, previous studies investigating ^111^In-labeled FibPep showed that the FibPep peptide was stable in serum, but prone to degradation in kidney and liver homogenates [16]. FibPep-ION-Micelles are, unlike the small ^111^In-labeled FibPep peptides, not expected to extravasate, encounter liver and kidney proteolytic enzymes and subsequently reenter the circulation. In addition, even though the ^111^In-labeled FibPep peptides were susceptible to degradation by liver and kidney homogenates, ^111^In-FibPep accumulated significantly more in carotid artery thrombi with respect to negative control peptide ^111^In-NCFibPep [16]. Therefore, instability of the targeting peptides on the FibPep-ION-Micelles is likely not a main factor for the identical level of uptake *in vivo* for the FibPep- and NCFibPep-ION-Micelles. Second, the lipidic micellular nanoparticle coating has a dynamic nature [20–22], possibly enabling fibrin-bound FibPep-ION-Micelles to dissociate from the fibrin target by shedding the fibrin-bound FibPep-lipid construct from the nanoparticle. However, *in vitro* blood clot binding experiments showed that the nanoparticles were bound to the clots in a stable fashion for more than one-and-a-half year. Third, serum proteins may form a protein corona [23], which could hinder targeting moieties on the nanoparticle surface to bind to their target [24]. However, the nanoparticles are virtually entirely coated with polyethylene glycol (PEG), which is known to mitigate the negative impact of the protein corona on nanoparticle targeting [25]. Fourth, nanoparticles accumulate in thrombi *in vivo* due to entrapment in the mesh-like clot structure [8,9]. This entrapment-based uptake might be less evident in the *in vitro* clot-binding experiments due to lack of blood flow, which launches nanoparticles into the thrombus mesh, and/or different morphologies of the thrombi *in vitro* and *in vivo*. Consequently, entrapment-based accumulation of FibPep-ION-Micelles and NCFibPep-ION-Micelles *in vivo* in the carotid artery thrombi may dominate the fibrin-specific uptake effects of the fibrin-binding FibPep-ION-Micelles.

In summary, the ION-Micelles significantly decreased thrombus T_2_ values and significantly increased MPS signal of the thrombi and thus were successfully able to detect thrombosis using MRI and MPS. The observed nanoparticle thrombus uptake is most likely due to entrapment-based accumulation of the nanoparticles in the thrombi *in vivo*. Such an entrapment-based strategy [8,9] using (nontargeted) ION-Micelles might be valuable for noninvasive MPI-based diagnosis, characterization and treatment monitoring of thrombosis. To further explore the potential of the ION-Micelle nanoplatform for MPI-based thrombosis diagnostics, preclinical *in vivo* MPI studies using a small animal MPI scanner are warranted. Such studies are well positioned to gauge sensitivity- and resolution-aspects of the proposed ION-Micelle thrombus diagnostic methodology.

Furthermore, since the FibPep-ION-Micelles did show specificity for fibrin *in vitro*, the FibPep-ION-Micelles might still find value for visualization of fibrin deposition in other pathologies, such as atherosclerosis, which do not involve large intraluminar mesh-like structures such as thrombi that may lead to high levels of nonspecific nanoparticle entrapment. Finally, the ION-Micelle platform shows high transversal relaxivity and strong MPS signal and allows conjugation to targeting ligands other than FibPep via facile maleimide-thiol chemistry, and is therefore a promising multi-purpose nanoplatform for molecular MRI and MPI strategies.

## Conclusions

ION-Micelles significantly decreased thrombus T_2_ values and significantly increased MPS thrombus signal in a carotid artery thrombosis mouse model. Functionalization of the ION-Micelles with fibrin-binding peptides did not result in a significant increased thrombus accumulation *in vivo*, indicating that entrapment of the nanoparticles in the thrombus-mesh is the chief contributor to *in vivo* nanoparticle thrombus uptake. All in all, the ION-Micelles showed high potential for noninvasive MPI-based diagnosis, characterization and treatment monitoring of thrombosis. Further studies investigating the potential of the ION-Micelle nanoplatform for *in vivo* MPI of thrombosis are therefore warranted.

## Experimental

### Materials

Materials were obtained from Sigma-Aldrich unless otherwise specified. Succinimidyl acetylthioacetate (SATA) was acquired from Invitrogen and Rink amide resin and 9-fluorenylmethyloxycarbonyl (Fmoc) protected amino acids were obtained from either Bachem or Novabiochem (Merck). 1,2-distearoyl-sn-glycero-3-phosphoethanolamine-N-[maleimide(polyethyleneglycol)-2000] (Mal-PEG200-DSPE) was purchased from Avanti Polar Lipids, 1,2-distearoyl-sn-glycero-3-phosphoethanolamine-N-[methoxy(polyethyleneglycol)-2000] (PEG200-DSPE) was acquired from Lipoid and near-infrared664-1,2-distearoyl-sn-glycero-3-phosphoethanolamine (NIR664-DSPE) was obtained from SyMO-Chem B.V. [26]. Citrated human plasma was purchased from Sanquin, human tissue factor was obtained from Dade Behring and Resovist was acquired from Bayer Schering Pharma.

### Nanoparticle synthesis and characterization

SATA-modified fibrin-binding peptide FibPep (Ac-RWQPCPAESWT-Cha-CWDPGGGK[SATA]-NH_2_) and a scrambled negative control peptide with C-A substitutions (NCFibPep, Ac-WPTAD-Cha-RAWPSQEWPAGGGK[SATA]-NH2_2_) were synthesized according to a previously published protocol [10]. ION-Micelles were synthesized and subsequently functionalized with (NC)FibPep peptides following a previously published procedure [10]. The final volume of the obtained (NC)FibPep-ION-Micelle solution was ca. 1 mL.

The nanoparticles were characterized using DLS, cryo-TEM, proton relaxometry (1.4 T, 37°C), MPS (30 s, 10 mT, 25 kHz), inductively coupled plasma atomic emission spectrometry (ICP-AES) and an *in vitro* blood clot binding assay (n = 2 per group, 35 μg Fe per sample) according to methods which were previously published [10]. After initial MPS measurements of the clots in the *in vitro* blood clot assay, the clots were stored at 4°C for more than 1.5 years. After this storage period, clots were again washed 3x with HBS pH 7.4, photographed and subjected to MPS.

### Animal model

All procedures regarding animals were approved by the ethical review committee of Maastricht University (permit: DEC2012-174) and were performed according to the Dutch national law and the guidelines set by the institutional animal care committee, accredited by the national department of health. All efforts were made to minimize suffering of the animals.

A well-established AlCl_3_-induced carotid artery thrombosis mouse model was chosen to study *in vivo* thrombus uptake of the nanoparticles [17]. C57BL/6 mice (Charles River Laboratories, 24.5 ± 1.6 g bodyweight) were housed under standard conditions with water and food freely available and acclimatized for at least one week before the start of the experimental procedures. Prior to thrombus inducing surgery, mice were subcutaneously injected with buprenorphine hydrochloride (Schering-Plough, 0.1 mg kg^-1^) for pain relief purposes. The mice were anesthetized using isoflurane, and a segment of the right carotid artery was surgically exposed. Wall-adherent thrombus formation was subsequently induced by applying a small piece of cleaning cloth soaked in 10% AlCl_3_ on the carotid for 5 min. Next, the cloth was removed, the carotid was washed with saline and the surgical wound was closed by a suture. Finally, a cannula filled with ca. 100 μL saline containing 50 U heparin mL^-1^ was connected to the tail vein to allow nanoparticle injection subsequent to the pre-injection MR scans without requiring repositioning of the mice. The nanoparticle bolus was contained in a second cannula which was attached to the first at the time of injection.

### *In vivo* MRI and *ex vivo* MPS of carotid thrombosis using (NC)FibPep-ION-Micelles

Mice were positioned in supine position into a 9.4 T MRI scanner equipped with a 35-mm-diameter volume transceiver coil (Bruker BioSpin GmbH) and were kept under continuous anesthesia using isoflurane (1–2%). Respiration frequency was monitored with a pressure balloon and temperature was maintained at 37°C with a heating pad and a rectal temperature probe for feedback. ECG signal was acquired by application of ECG paste on the front paws of the mice, which were subsequently positioned onto gold-plated ECG electrodes that were connected to an ECG triggering system (Small Animal Instruments Inc).

The MRI protocol consisted of a scan of the chest and neck region with a 3D-FLASH-TOF acquisition (echo time TE = 2.5 ms, repetition time TR = 17 ms, field of view FOV = 20 × 20 × 25.6 mm^3^, matrix = 200 × 200 × 256, number of averages NA = 2, flip angle FA = 20°). This 3D dataset was used to plan 13 parallel slices (thickness = 0.5 mm, slice separation 0.1 mm, FOV = 25.6 × 25.6 mm^2^, matrix = 256 × 256) perpendicular to the right carotid artery, such that the last slice was positioned distal to the bifurcation (Fig. 3B). Subsequently, T_1_- and T_2_-weighted multi slice spin echo (NA = 2, T_1_: TE/TR = 7.5/800 ms, T_2_: TE/TR = 20/2000 ms) images were acquired in this slice geometry. The center slice in the thrombus was chosen for T_2_ mapping which was performed with an ECG-triggered, respiratory gated, segmented, MLEV-prepared scan with fast imaging with steady-state precession (FISP) readout [27] (40 segments of 5 echoes, TE = 2.1 ms, TR = 4.1 ms, segment TR = 2000 ms, NA = 4, FA = 30°, FOV 20 × 20 mm^2^, matrix 400 × 400, zero-filling 2 × 2). Images with six effective echo times (TE_eff_ = 0.2, 7.0, 14.6, 21.0, 29.0 and 35.4 ms) were acquired.

The full MRI protocol was performed twice in the same scan session. In between, at ca. 2.5 h post-thrombus formation, FibPep-ION-Micelles or NCFibPep-ION-Micelles (100 μL, 175 μg Fe, n = 5 per group) were injected together with the saline already present in the cannula. To this end, the cradle containing the mouse had to temporarily be removed out of the bore of the MRI scanner. By leaving the mouse fixed in the cradle its orientation was largely preserved. However, to prevent repositioning errors the MRI slice planning was repeated. The T_1_- and T_2_-weighted images and the T_2_ map were acquired at approximately 49, 62 and 94 min post-injection, respectively. After the final MR scans (ca. 2 h post-nanoparticle injection), mice were euthanized by incision of the diaphragm and the vena cava. The injured and contralateral carotids were subsequently harvested and subjected to MPS measurements (10 mT, 25 kHz, 30 s, RT). Additionally, the injured and contralateral carotids of three mice, which did undergo thrombus inducing surgery but no nanoparticle injection, were excised and measured with MPS.

### MRI data analysis

Image analysis was performed using a custom-built algorithm in Mathematica 9.0 (Wolfram Research). A region of interest (ROI) was manually drawn around the right carotid artery with thrombus on one of the T_2_-weighted images of the pre-injection T_2_ map. The center of the artery was marked and used as a landmark to register pre- and post-injection images. The registration was visually inspected and corrected manually if necessary. The validity of the ROIs was additionally assessed by registration to the multi-slice T_1_- and T_2_-weighted images and visual inspection. Subsequently, pixel-wise mono-exponential fitting of the signal intensities at different TE_eff_ was performed. Next, the mean T_2_ value of the ROI was determined, and pixels with an R^2^ of fit < 0.7 were excluded from further analysis.

### Histology

After the MPS measurements, the injured and contralateral carotid arteries were embedded in Tissue-Tek matrix (Sakura) and subsequently snap-frozen in isopentane and stored at -80°C. The arteries were cut in transversal sections of 5 μm and covered with Fluoromount and a cover glass. Confocal fluorescence images were recorded at RT on a Leica TCS SP5 system (Leica Microsystems). Autofluorescence was measured using a 488 nm argon laser and an emission window of 530–580 nm.

### Statistical analysis

All data represent the mean value ± standard deviation (SD). For differences between more than two groups, a 1-way ANOVA with Bonferroni’s multiple comparison procedure was employed. Group normality was tested with a Shapiro-Wilk test and Levene’s test was used to assess the equality of variances between groups. Comparison of FibPep- and NCFibPep-ION-Micelles MRI data was done using a two-sided independent t-test and pre- and post-injection data were compared with a two-sided paired t-test. For all statistical analysis, values of p < 0.05 were considered to be significant.

## Supporting Information

S1 Fig *In vitro* ION-Micelle human plasma clot binding. (A) Photograph and (B) MPS of human plasma clots incubated with either FibPep-ION-Micelles (n = 2) or NCFibPep-ION-Micelles (n = 2). MPS measurements were performed immediately after the incubation and washing procedure (Day 0) and after storage for more than 1.5 years (> 1.5 years). Data is expressed as magnetic moment of the third harmonic (76 kHz).
(TIF)
