# Supplementary figures and images for: Evaluation of Iron Oxide Nanoparticle Micelles for Magnetic Particle Imaging (MPI) of Thrombosis
Source: PLoS One. 2015 Mar 6;10(3):e0119257. doi: 10.1371/journal.pone.0119257 (PMC4352001; doi:10.1371/journal.pone.0119257)

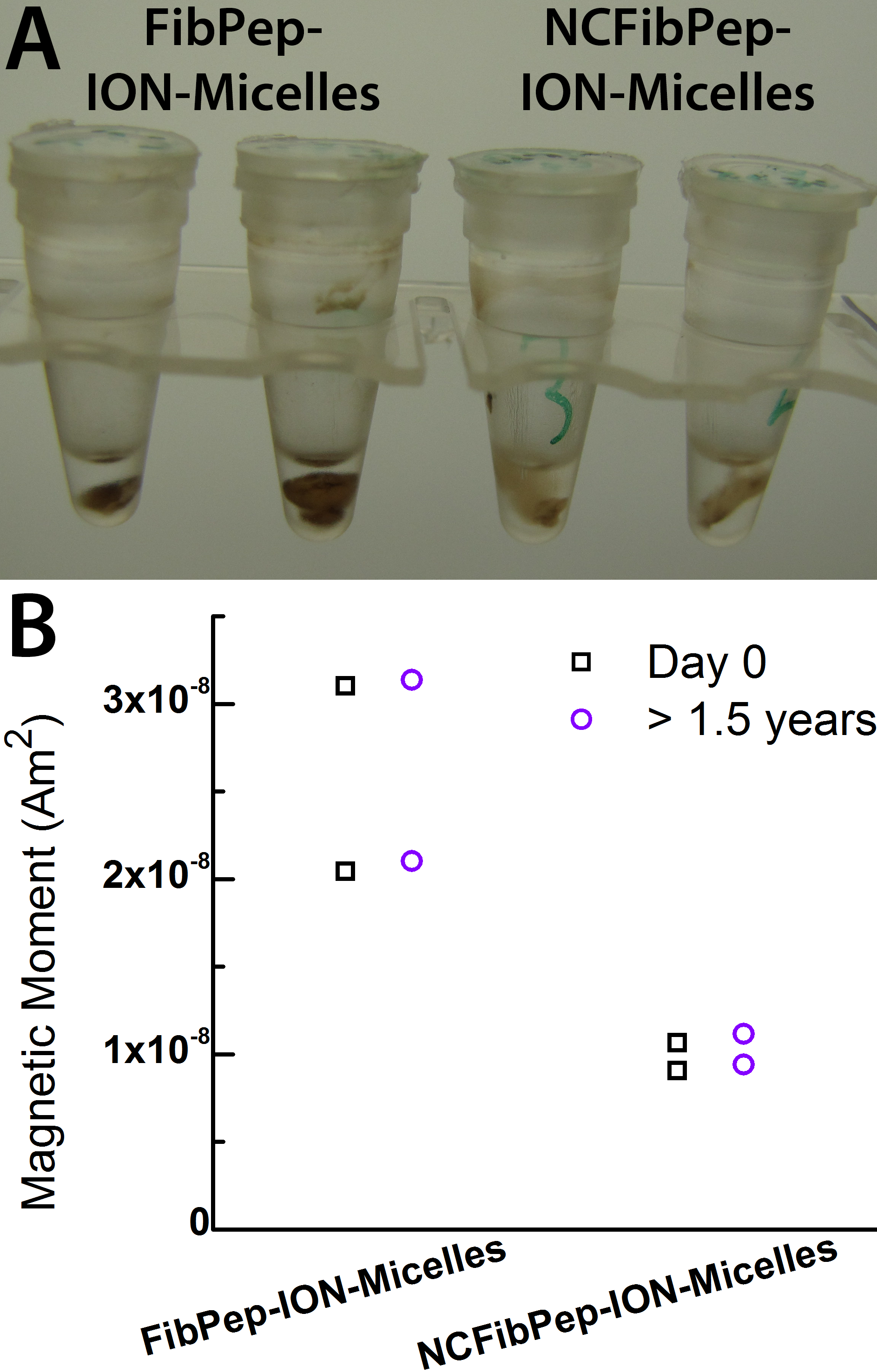

Supplement: S1 Fig — (A) Photograph and (B) MPS of human plasma clots incubated with either FibPep-ION-Micelles (n = 2) or NCFibPep-ION-Micelles (n = 2). MPS measurements were performed immediately after the incubation and washing procedure (Day 0) and after storage for more than 1.5 years (> 1.5 years). Data is expressed as magnetic moment of the third harmonic (76 kHz). (TIF) [file pone.0119257.s001.tif]
